# Current trends and challenges: The landscape of perioperative mortality in intracranial surgeries in low‐ and middle‐income settings: A narrative review

**DOI:** 10.1002/hsr2.1838

**Published:** 2024-01-25

**Authors:** Sakshi Roy, Wireko Andrew Awuah, Arjun Ahluwalia, Favour T. Adebusoye, Tomas Ferreira, Joecelyn K. Tan, Hareesha R. Bharadwaj, Pearl O. Tenkorang, Toufik Abdul‐Rahman, Marios Papadakis

**Affiliations:** ^1^ School of Medicine Queen's University Belfast Belfast UK; ^2^ Faculty of Medicine Sumy State University Sumy Ukraine; ^3^ Department of Clinical Neurosciences, School of Clinical Medicine University of Cambridge Cambridge UK; ^4^ Faculty of Medicine University of St Andrews St. Andrews UK; ^5^ Faculty of Biology, Medicine and Health The University of Manchester Manchester UK; ^6^ Faculty of Medicine University of Ghana Accra Ghana; ^7^ Department of Surgery II, University Hospital Witten‐Herdecke University of Witten‐Herdecke Wuppertal Germany

**Keywords:** global neurosurgery, intracranial surgeries, low‐ and middle‐income countries, perioperative mortality

## Abstract

**Background and Aims:**

Intracranial surgeries are pivotal in treating cerebral pathologies, particularly in resource‐limited contexts, utilizing techniques such as craniotomy, transsphenoidal approaches, and endoscopy. However, challenges in low and middle income countries (LMICs), including resource scarcity, diagnostic delays, and a lack of skilled neurosurgeons, lead to elevated perioperative mortality (POM). This review seeks to identify major contributors to these challenges and recommend solutions for improved patient outcomes in neurosurgical care within LMICs.

**Methods:**

This review examines POM in LMICs using a detailed literature search, focusing on studies from these regions. Databases like PubMed, EMBASE, and Google Scholar were utilized using specific terms related to “intracranial surgery,” “perioperative mortality,” “traumatic brain injuries,” and “LMICs.” Inclusion criteria covered various study designs and both pediatric and adult populations while excluding stand‐alone abstracts and case reports.

**Results:**

POM rates for intracranial surgeries differ widely across many low and middle‐income regions: Africa sees rates from 2.5% to 39.1%, Asia between 3.6% and 34.8%, and Latin America and the Caribbean have figures ranging from 1.3% to 12%. The POM rates in LMICs were relatively higher compared to most first‐world countries. The high POM rates in LMICs can be attributed to considerable delays and compromises in neurosurgical care delivery, exacerbated by late diagnoses and presentations of neurosurgical pathologies. This, coupled with limited resources, underdeveloped infrastructure, and training gaps, complicates intracranial disease management, leading to elevated POM.

**Conclusion:**

Intracranial POM is a pronounced disparity within the neurosurgical field in LMICs. To mitigate intracranial POM, it is imperative to bolster healthcare infrastructure, amplify personnel training, foster global partnerships, and harness technologies like telemedicine. Tackling socioeconomic obstacles and prioritizing early detection through sustained funding and policy shifts can substantially enhance patient outcomes.

## 1 BACKGROUND

Intracranial surgical interventions, encompassing life‐saving procedures performed within the skull, are important interventions used to address a broad spectrum of cerebral pathologies. These surgeries employ an array of techniques, including but not limited to, craniotomy, transsphenoidal approaches, endoscopic procedures, stereotactic surgery, shunting procedures, microvascular decompression, and awake craniotomy. Each method is applied with the intent to mitigate symptoms, improve patient's quality of life, and ultimately prolong survival.^1^, ^2^, ^3^

Perioperative mortality (POM), which refers to deaths within 30 days postsurgery, remains a significant concern, especially in low‐ and middle‐income countries (LMICs). The prevalence of intracranial pathologies, including traumatic brain injuries (TBI), brain tumors, vascular malformations, and epilepsy, increases mortality risk in these regions.^1^, ^4^, ^5^, ^6^ Notably, TBI is a leading cause of death and disability globally, imposing a disproportionate burden on LMICs. TBI incidence rates in LMICs exceed the global average, with inhabitants of these regions facing twice the risk of death following severe TBI in comparison to individuals in high‐income countries (HICs).^7^, ^8^

Addressing POM in LMICs poses several challenges. Limited access to healthcare, socioeconomic disparities, and inadequate preventive measures collectively contribute to the increased demand for intracranial surgeries in LMICs.^4^, ^6^, ^9^ The provision of comprehensive surgical care for intracranial diseases within these countries encounters obstacles such as limited availability of essential resources and diagnostic technologies. For instance, HICs boast a significantly higher per capita ratio of computed tomography (CT) scanners, contributing to diagnostic delays in intracranial hemorrhage identification and subsequent treatment in LMICs.^10^ Additionally, the scarcity of trained neurosurgeons is strikingly apparent in LMICs, with the deficit most pronounced in Africa.^9^, ^11^ The notably fewer neurosurgeons per capita in LMICs result in delays and compromised delivery of neurosurgical care. Compounding these issues, the late presentation of neurosurgical pathologies complicates intracranial disease management, subsequently increasing morbidity and mortality rates.^4^, ^6^, ^11^, ^12^ These challenges in LMICs are amplified by limited resources, underdeveloped infrastructure, and the lack of educational materials and training programs. Such limitations restrict the timely and appropriate execution of surgical interventions, thereby exacerbating POM associated with intracranial surgeries.^4^, ^6^, ^9^, ^11^, ^12^

Furthermore, limited follow‐up care and substandard preoperative preparation contribute to the management gaps of intracranial surgeries in LMICs. Inadequate postoperative monitoring and access to rehabilitation services often lead to suboptimal recovery and an increased risk of complications.^4^, ^6^, ^12^ Similarly, poor preoperative evaluation and preparation, including comprehensive patient assessment, and exhaustive preoperative imaging, pose further challenges to intracranial surgeries in LMICs.^4^, ^6^, ^12^

In light of challenges, this review aims to highlight the principal contributors to POM in intracranial surgeries within LMICs and offer recommendations to address these challenges, fostering improved patient outcomes, enhanced safety measures, and equitable access to high‐quality neurosurgical care.

## 2 METHODOLOGY

This narrative review delves into the landscape of POM within LMICs, employing a rigorous methodology characterized by a comprehensive search of published literature, with a specific focus on studies conducted in low and middle income settings. The detailed search string, as provided in the appendix, facilitated an extensive and targeted exploration of the available literature.

Inclusion criteria were designed to encompass studies of various designs, including observational, case‐control, cohort, and randomized controlled trials. This review considered studies involving both pediatric and adult populations. Strict adherence to English language publications was maintained, and a defined time frame was imposed to align with current practices, thereby enhancing the validity and relevance of the findings. Stand‐alone abstracts, unpublished studies, non‐English studies, and case reports were excluded. The reviewed studies revolved around patients who had undergone surgery for intracranial pathologies, as previously discussed.

To conduct the literature search, databases such as PubMed, EMBASE, and Google Scholar were meticulously employed. A set of precise search terms, which encompassed “perioperative,” “intracranial surgery,” “mortality,” “traumatic brain injury,” “brain tumors,” “vascular malformations,” and “epilepsy,” were coupled with geographical identifiers including “LMICs,” “low and middle income countries,” and “low and middle income settings.” This approach ensured that the literature search was tailored to our specific area of interest. Furthermore, a manual search was executed to identify references related to recently published, procedure‐specific reviews that could further enhance our understanding of the LMIC context. Stand‐alone abstracts and unpublished studies were deliberately excluded from the review.

Through this comprehensive and meticulous approach, the review endeavors to furnish a high‐quality scholarly assessment of POM in LMICs. It aims to provide an in‐depth synthesis of pertinent findings, offering insights that may have broader applicability in similar income settings. A summary of the methodology has been presented in Table 1.

**Table 1:** Summary of the methodology employed in the study.

Methodology steps	Description
Literature search	PubMed, EMBASE, Google Scholar, the Cochrane Library, EMBASE, CINAHL, SCOPUS, Scielo (English only).
Inclusion criteria	Full‐text articles published in English. Various study designs, such as observational, case‐control, cohort, cross‐sectional, and randomized controlled trials. Studies involving pediatric and adult populations. Studies addressing POM from intracranial pathology.
Exclusion criteria	Stand‐alone abstracts and unpublished studies. Non‐English studies, case reports.
Search terms	Keywords include “perioperative,” “intracranial surgery,” “mortality,” “traumatic brain injury,” “brain tumors,” “vascular malformations,” and “epilepsy,” were coupled with geographical identifiers including “LMICs,” “low and middle income countries,” and “low and middle income settings.”
Additional search	A manual search was conducted to find references for recently published, procedure‐specific reviews.

## 3 POM RATES (POMR) IN LMICS: A REPORT OF GLOBAL INCIDENCE

Data on the global prevalence of POMR in LMICs is sparse. POMRs exhibit substantial variability across LMICs, contingent upon the specific surgical procedures and diseases under investigation. In the African context, the literature reveals a broad spectrum of POMRs, with rates ranging between 2.5% and 39.1% for an assortment of intracranial interventions and pathologies.^1^, ^4^, ^13^, ^14^, ^15^, ^16^, ^17^, ^18^ This stands in sharp contrast to the trends observed in North America and Europe, where analogous intracranial surgical interventions have consistently manifested markedly reduced POMRs.^19^, ^20^, ^21^, ^22^, ^23^ Notably, certain studies within these developed regions have even reported an absence of perioperative mortalities.^21^, ^22^, ^23^

Transitioning to the Asian context, the reported POMRs for a diverse range of surgical interventions and associated diseases span between 3.6% and 34.8%.^2^, ^3^, ^6^, ^12^, ^24^, ^25^, ^26^ Latin America and the Caribbean, intriguingly, have recorded relatively modest POMRs in comparison to other LMIC regions. This observation is corroborated by seminal works such as those by Pereira et al. and Celi et al., wherein the documented POMRs fluctuated between a mere 1.3% and 12% contingent upon the specific surgical modalities and diseases.^5^, ^27^

In a nuanced study from Eastern Europe, Dzhindzhikhadze et al. presented an evaluation of the mini zygomatic craniotomy—a specialized surgical technique. Their findings underscored a POMR of 3.3% associated with this procedure, which was employed to address a myriad of conditions.^28^

## 4 RISK FACTORS ASSOCIATED WITH POM DURING INTRACRANIAL SURGERIES IN LMICS

The risk factors implicated in POM associated with intracranial surgeries are multifactorial, spanning elements pertinent to the patient, surgical procedures, and the healthcare system. The identification of these risk factors is crucial for improving patient outcomes and reducing mortality rates within LMICs.

### 4.1 Patient‐related factors

Several patient‐related factors have been associated with an increased risk of intracranial POM in LMICs. These factors include hypertension, diabetes, use of antiplatelets and anticoagulants, alcohol abuse, hematoma laterality, financial constraints, poor acceptance due to ignorance, cultural beliefs, age, gender, preoperative neurological status, altered sensorium, Karnofsky performance status score, and prior treatment history.^1^, ^2^, ^3^, ^4^, ^13^, ^14^, ^15^, ^16^, ^17^ The interplay of these factors has significant potential to impact the overall outcome of intracranial surgeries and consequently contribute to POM.

In a study by Eaton et al. in Malawi, advanced age and lower Glasgow coma scale scores were associated with increased mortality rates in patients undergoing exploratory burr hole procedures for TBI.^13^ The study further noted that survivors were more likely to have been subjected to exploratory burr hole procedures, implying the potential influence of these procedures on patient outcomes.

Similarly, Laeke et al. identified patient‐related factors like diabetes mellitus, hypertension, and bronchial asthma, in addition to tumor‐related determinants such as size and location, as influencers of POM in patients with intracranial meningiomas.^1^ Acknowledging and addressing these factors is integral to improving patient outcomes and effectively managing such cases.

### 4.2 Surgical and tumor characteristics

Several potential risk factors have been identified for POM in intracranial surgeries in LMICs, encompassing both surgical and tumor‐related factors. Surgical factors, such as surgery location (infra‐ or supratentorial), urgency of the procedure, surgery duration, head shaving, application of wound drains, intraoperative ultrasound, and extent of resection, have been identified as critical.^1^, ^24^ Furthermore, the choice of surgical approach and technique has a significant bearing on the outcome of intracranial surgeries. Moiyadi et al. underscored that factors such as the location of the tumor, the emergent nature of the surgery, and the extent of resection have a substantial influence on morbidity and mortality rates in intra‐axial brain tumor surgeries within LMICs.^24^

Additionally, tumor‐associated characteristics, including size, location, and histology, have been implicated in POM for intracranial surgeries.^1^, ^24^ For instance, studies have demonstrated that tumors exceeding 5 cm in size and those in specific locations are associated with increased mortality in patients with intracranial meningiomas.^1^ Understanding these tumor characteristics is vital to selecting the appropriate surgical approach and accurately predicting outcomes.

## 5 EXPLORING GAPS IN THE MANAGEMENT OF INTRACRANIAL SURGERIES CONTRIBUTING TO POM IN LMICS

### 5.1 Healthcare infrastructure and resource disparities

A robust healthcare infrastructure and adequate resources are vital for intracranial surgery to ensure patient safety, facilitate accurate diagnosis and surgical planning, and provide comprehensive care throughout the surgical process. However, these are not readily available in LMICs.^3^, ^4^ In particular, LMICs face challenges such as a scarcity of essential equipment, inadequate aftercare facilities, a lack of neurointensive care units, limited availability of medications, and the threat of hardware failures and power outages. The limited availability of these vital tools significantly hampers the provision of adequate care, further increasing the risk of complications and mortality during or after the surgical procedure. Rock et al. delineated the correlation between heightened mortality rates and restricted access to vital resources such as intracranial pressure monitoring, ventilators, and electroencephalography.^6^ This trend is observed across multiple LMICs, indicating a prevalence of suboptimal healthcare infrastructure for intracranial surgery in these regions.^2^, ^3^, ^4^, ^16^, ^27^

### 5.2 Financial and socioeconomic challenges

The financial and socioeconomic implications of intracranial surgery extend beyond patient outcomes, impacting long‐term healthcare expenditures and overall societal productivity. However, within LMICs, financial and socioeconomic factors have been identified as significant contributors to intracranial mortality.^1^, ^14^ The complex challenges faced by these nations include limited insurance coverage, significant out‐of‐pocket costs, inadequate financial support programs, and the absence of reimbursement systems for affordable intracranial surgery services.^13^, ^27^ The study conducted by Uche et al. describes how financial limitations can pose significant barriers for patients requiring novel surgical interventions such as neuroendoscopic procedures.^4^ This trend is pervasive across numerous LMICs, where such limitations contribute to suboptimal preoperative optimization and substandard postoperative care and monitoring, resulting in increased POM.^6^, ^13^

### 5.3 Inadequate neurosurgical expertise and limited multidisciplinary team involvement

Neurosurgical expertise and other MDTs are crucial for the effective management of various conditions and procedures. However, LMICs continue to face significant challenges, including lack of knowledge and experience in managing intracranial lesions, inability to execute systematic therapeutic choices (e.g., exploratory burr hole), inadequate staffing, learning curve‐related complications in novel neurosurgical procedures, and the lack of guidelines implementation for complex neurosurgical procedures.^2^, ^16^ For instance, Fatigba et al. underscored the detrimental effect of inadequate intracranial lesion knowledge of mortality rates in the Benin Republic.^16^ Additionally, Rehman et al. emphasized the shortage of experienced neurosurgeons and endocrinologists in Pakistan, coupled with ineffective interspecialty communication.^3^ Thus, the effective management of intracranial surgeries in LMICs continues to be impeded by the aforementioned limitations.

### 5.4 Research gaps and ethical considerations

Research and ethical considerations are important in intracranial surgery in LMICs to prioritize patient safety, optimize resource use, and ensure the well‐being of surgical patients. However, a prominent research gap persists in LMICs, attributable to the paucity of research experience and the absence of projects focusing on neurosurgical care.^4^ The lack of research hinders the understanding of effective POMR management strategies and impedes the development of evidence‐based practices.^29^

Additionally, the absence of electronic medical records in LMICs further complicates research efforts. The absence of standardized data collection and storage systems curtails the capacity for comprehensive studies and the compilation of valuable data for analysis and improvement.^6^ Implementation of electronic medical records could bolster research capabilities, enable data‐driven decision‐making, and contribute to advancements in neurosurgical care within LMICs. Furthermore, the lack of comprehensive follow‐up and long‐term outcome assessments introduces ethical concerns regarding patient care and informed decision‐making. Limited access to follow‐up care and the inability to assess long‐term outcomes hinder the ability to accurately monitor patient progress. Ethical considerations arise in balancing immediate interventions with long‐term patient well‐being and ensuring informed decision‐making.^30^

**Figure 1:**
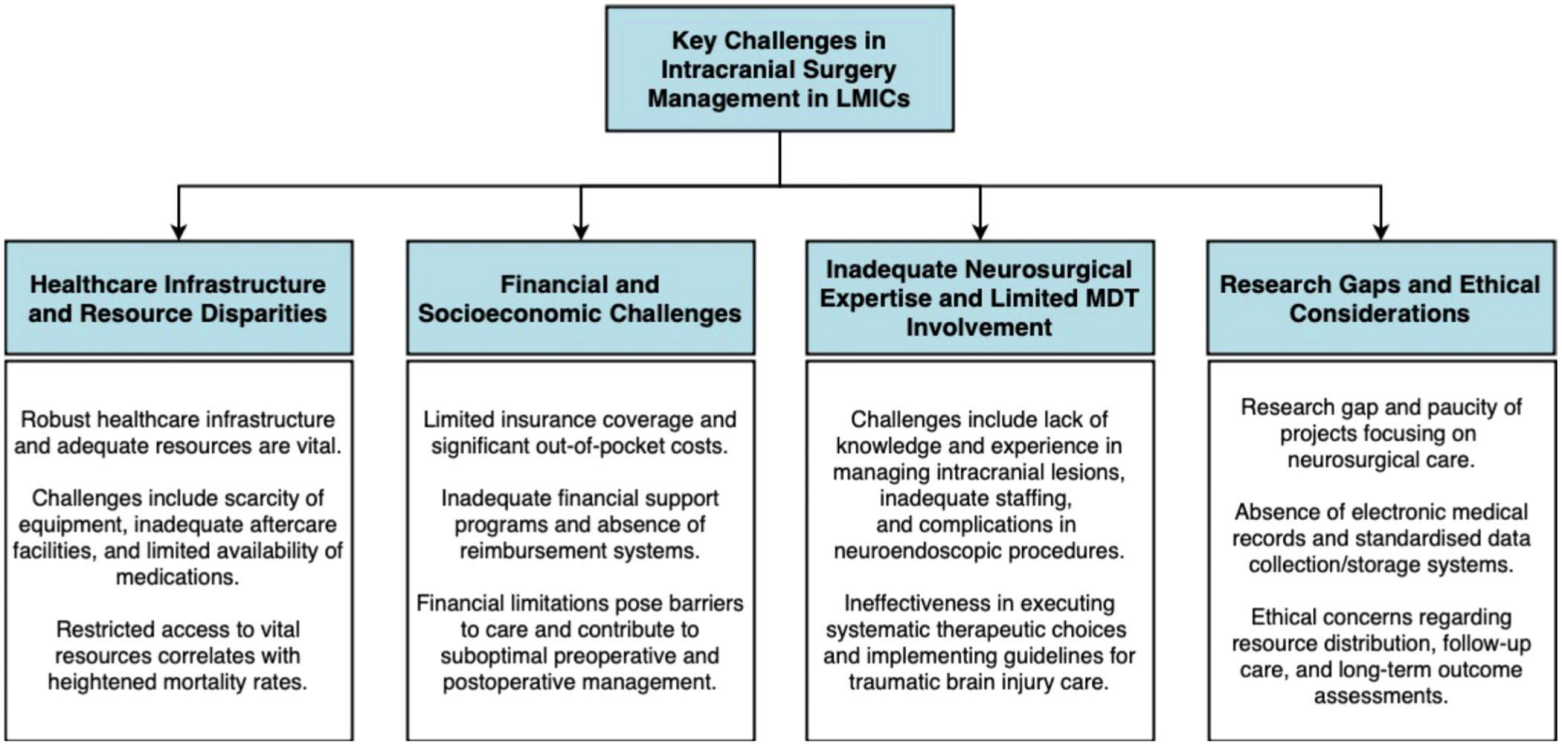
A summary of the discussion on key challenges in intracranial surgery management faced in low and middle‐income countries (LMICs). MDT, multidisciplinary team.

These studies collectively underscore the multifaceted nature of intracranial mortality in LMICs and the need for comprehensive strategies to address healthcare infrastructure deficits, financial barriers, inadequate neurosurgical expertise, limited multidisciplinary team involvement, and research gaps for effective management of intracranial conditions in LMICs. A summary of the above discussion has been provided in Figure 1.

## 6 LOWERING POMRS IN INTRACRANIAL SURGERIES IN LMICS: STRATEGIC INTERVENTIONS

To address the pressing issue of perioperative intracranial mortality in LMICs, an approach involving the concerted efforts of policymakers, healthcare providers, and researchers is recommended. This approach aims to enhance healthcare systems, improve surgical competence and safety, foster collaborative partnerships, and leverage technology and innovation. For example, an effective solution lies in the promotion of trained healthcare personnel, which ensures access to essential resources and improved patient outcomes.^9^, ^11^, ^31^ This requires policymakers to enact schemes facilitating specialized training, workshops, and conferences to develop highly competent neurosurgeons and reduce the POM associated with intracranial surgeries.

Strengthening healthcare systems in LMICs necessitates ensuring adequate resources for vital infrastructure, including operating rooms, imaging facilities, and essential neurological instruments.^11^, ^32^ Enhancing surgical skills and safety through the adoption of standardized protocols and guidelines is paramount. This can be achieved by fostering professional development, conducting regular audits to pinpoint areas of improvement, and investing in research to formulate evidence‐based practices. Notably, training programs emphasizing the technical facets of surgical interventions, such as neuroendoscopy, have been instrumental in mitigating postoperative complications.^4^

Neurosurgical advancements in LMICs have greatly benefited from international collaborations. These alliances, especially with prominent global institutions, provide crucial monetary support, resources, and skill enhancement. One significant example is the introduction of awake craniotomy techniques to LMICs, resulting in improved perioperative outcomes and shorter hospital stays.^32^ Furthermore, these international partnerships have been instrumental in augmenting neurosurgical management in LMICs by facilitating fund flow and bolstering healthcare infrastructure.^1^, ^13^, ^32^ They not only streamline financial frameworks but also introduce advanced educational training. Patient education, shared decision‐making, and sustained communication further strengthen this collaborative model. Although organizations like the World Federation of Neurosurgical Societies and the Foundation for International Education in Neurological Surgery have made strides in improving POMRs through knowledge and skill transfer,^31^ consistent funding remains a challenge. Here, enduring international collaborations are paramount, serving as essential funding networks to elevate POMRs in LMICs.

In LMIC neurosurgical settings, addressing financial barriers is paramount to achieving equitable healthcare access.^33^ Policymakers should devise fiscal strategies to alleviate neurosurgical healthcare costs, ensuring equitable access across socioeconomic lines. Investing in improved transportation infrastructure can expedite patient journeys to neurosurgical centers, reducing surgical delays, and alleviating financial burdens.^11^ Socioeconomic factors significantly impact health outcomes, such as perioperative intracranial mortality, and can impede early abnormality detection and prompt neurosurgical intervention. Laeke et al. emphasized the correlation between larger tumor sizes, specific tumor locations, and increased mortality rates, underscoring the imperative of early detection.^1^

**Figure 2:**
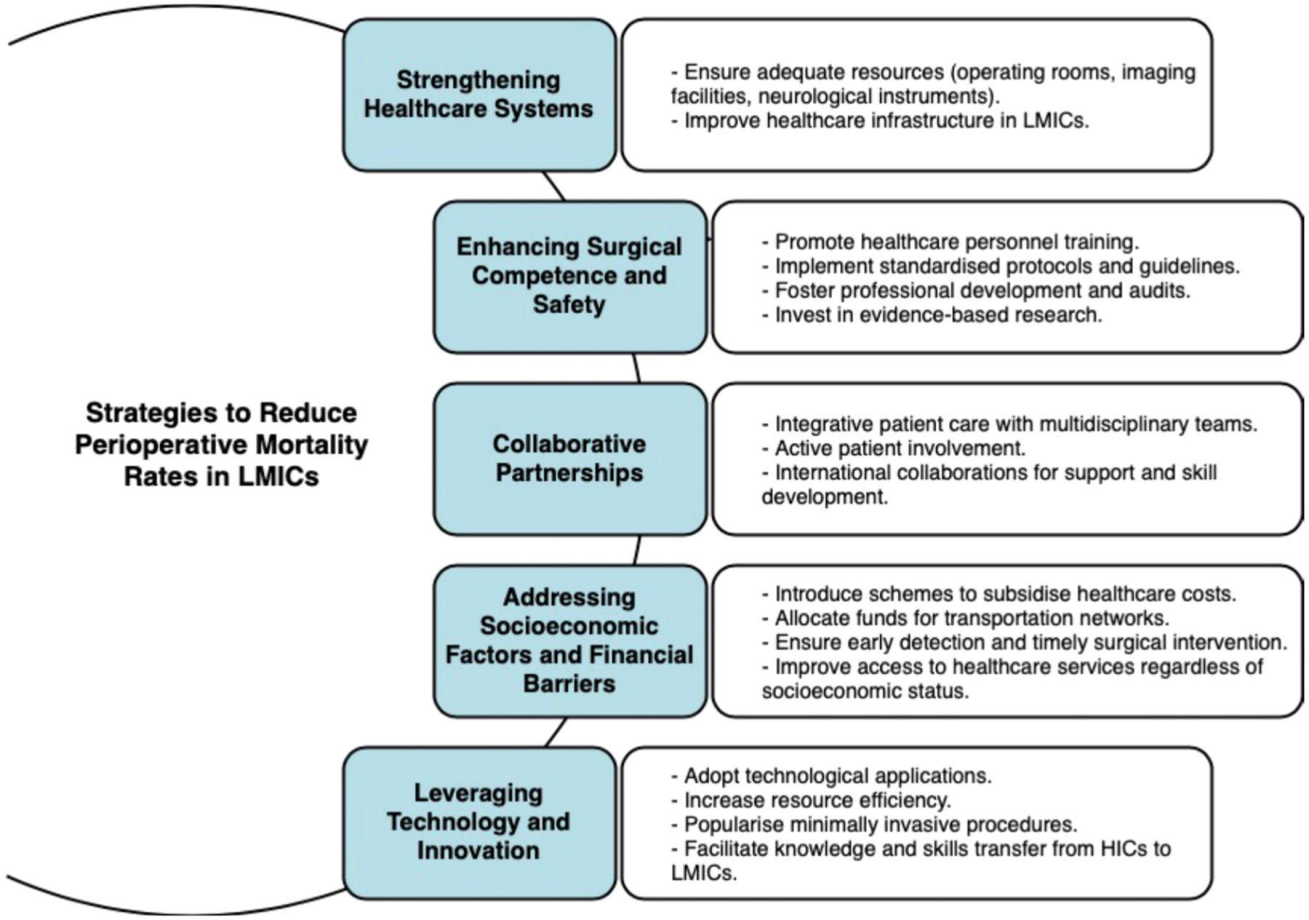
A discussion summary of strategies to reduce intracranial mortality in low and middle‐income countries (LMICs).

Amid the rapid technological adoption spurred by the COVID‐19 pandemic, the incorporation of innovations like telemedicine, mobile health platforms, eLearning, and surgical advancements has become crucial for neurosurgical practices in LMICs. These integrations enhance resource efficiency, empower patients, and facilitate continuous knowledge and skill transfer.^34^, ^35^ The rise of minimally invasive neurosurgical methods, notably neuroendoscopy, offers potential to reduce complications and optimize postoperative care.^36^ Nevertheless, it remains indispensable for neurosurgeons in LMICs to be endowed with meticulous training and unimpeded access to requisite surgical apparatus for these procedures. The schematic delineation of strategies geared toward the attenuation of intracranial mortality in LMICs is elucidated in Figure 2.

## 7 LIMITATIONS OF STUDY

The study's robust methodology notwithstanding, it is imperative to acknowledge and delineate several limitations. First, the constrictions placed on the literature search, confined solely to three databases—PubMed, EMBASE, and Google Scholar—could potentially entail the exclusion of pertinent articles disseminated through alternative sources. Additionally, the choice of specific keywords could have inadvertently omitted valuable literature due to the varied use of terminology.

A significant limitation arises from language bias, stemming from the exclusive scrutiny of English‐language publications. This restriction may have narrowed the spectrum of perspectives under consideration, potentially resulting in the omission of valuable insights from non‐English sources. Future research and reviews could seek to overcome this by including non‐English publications, implying the need for an extended search strategy, the incorporation of non‐English databases, and collaborations with experts in non‐English literature.

Furthermore, publication bias may have exerted an impact on the representation of surgical procedure mortality rates. Studies characterized by higher mortality rates might have encountered reticence in publication due to concerns regarding reputation, while those marked by lower mortality rates might have been deemed inconsequential for dissemination. Such bias introduces the potential for an incomplete comprehension of the mortality risks associated with diverse surgical procedures and could result in an underestimation of actual mortality rates. Another noteworthy limitation relates to the exclusion of neurosurgical studies that do not pertain to intracranial surgeries, such as spinal surgery. This selective focus might have curtailed the breadth of insights gleaned regarding POM in the context of neurosurgery within LMICs.

The presence of disparities in the definitions and criteria applied to POMR across different institutions within LMICs constitutes a notable limitation. As underscored by Ng‐Kamstra et al., the absence of a standardized approach for reporting POMRs in LMICs gives rise to substantial variations across distinct procedures and specialties. Such discrepancies have the potential to significantly affect the accuracy and reliability of the findings derived from the studies included in this review.^37^

## 8 CONCLUSION

POM in intracranial surgeries remains a significant concern, particularly in LMICs. Patient‐related factors, healthcare system challenges, and surgical and tumor‐related factors contribute to the increased mortality risk in LMICs. The situation is further exacerbated by financial limitations, scarce resources, a lack of neurosurgical expertise, and identified gaps in research. Addressing these challenges requires comprehensive and multifaceted approaches, including improvements to healthcare infrastructure, efforts to address financial and socioeconomic barriers, fostering multidisciplinary team involvement, and promoting research. Implementing these strategies can foster improved patient outcomes, enhance safety measures, and ensure equitable access to high‐quality neurosurgical care within LMICs. Additionally, the fostering of collaborative efforts and the sharing of knowledge within the global neurosurgical community could prove pivotal in addressing the mortality associated with intracranial surgeries in resource‐constrained environments.

## AUTHOR CONTRIBUTIONS

**Sakshi Roy**: Conceptualization; data curation; methodology; writing—original draft; writing—review and editing. **Wireko Andrew Awuah**: Conceptualization; data curation; methodology; validation; writing—original draft; writing—review and editing. **Arjun Ahluwalia**: Writing—original draft; writing—review and editing. **Favour T. Adebusoye**: Methodology; writing—original draft; writing—review and editing. **Tomas Ferreira**: Writing—original draft; writing—review and editing. **Joecelyn K. Tan**: Writing—original draft; writing—review and editing. **Hareesha R. Bharadwaj**: Formal analysis; methodology; writing—original draft; writing—review and editing. **Pearl O. Tenkorang**: Investigation; methodology; writing—original draft; writing—review and editing. **Toufik Abdul‐Rahman**: Investigation; methodology; writing—original draft; writing—review and editing. **Marios Papadakis**: Formal analysis; investigation; methodology; supervision. All authors have read and approved the final version of the manuscript.

## CONFLICT OF INTEREST STATEMENT

The authors declare no conflict of interest.

## TRANSPARENCY STATEMENT

The lead author Wireko Andrew Awuah affirms that this manuscript is an honest, accurate, and transparent account of the study being reported; that no important aspects of the study have been omitted; and that any discrepancies from the study as planned (and, if relevant, registered) have been explained.

## Data Availability

No data associated with the article. Wireko Andrew Awuah had full access to all of the data in this study and takes complete responsibility for the integrity of the data and the accuracy of the data analysis.
